# Selected Tea and Tea Pomace Extracts Inhibit Intestinal α-Glucosidase Activity *in Vitro* and Postprandial Hyperglycemia *in Vivo*

**DOI:** 10.3390/ijms16048811

**Published:** 2015-04-21

**Authors:** Jungbae Oh, Sung-Hoon Jo, Justin S. Kim, Kyoung-Soo Ha, Jung-Yun Lee, Hwang-Yong Choi, Seok-Yeong Yu, Young-In Kwon, Young-Cheul Kim

**Affiliations:** 1 Department of Nutrition, University of Massachusetts, Amherst, MA 01003, USA; E-Mails: joh@umass.edu (J.O.); sunghoon04@hanmail.net (S.-H.J.); jsk2995@gmail.com (J.S.K.); kengkoo@nate.com (K.-S.H.); seokyeong@nutrition.umass.edu (S.-Y.Y.); 2 Department of Food and Nutrition, Hannam University, Daejeon 305-811, Korea; E-Mails: seembeeks@hanmail.net (J.-Y.L.); kelolo123@nate.com (H.-Y.C.)

**Keywords:** α-glucosidase, sucrase, inhibitor, postprandial hyperglycemia, pomace, green tea, oolong tea, black tea, diabetes

## Abstract

Type 2 diabetes mellitus (T2DM) is a metabolic disorder characterized by postprandial hyperglycemia, which is an early defect of T2DM and thus a primary target for anti-diabetic drugs. A therapeutic approach is to inhibit intestinal α-glucosidase, the key enzyme for dietary carbohydrate digestion, resulting in delayed rate of glucose absorption. Although tea extracts have been reported to have anti-diabetic effects, the potential bioactivity of tea pomace, the main bio waste of tea beverage processing, is largely unknown. We evaluated the anti-diabetic effects of three selected tea water extracts (TWE) and tea pomace extracts (TPE) by determining the relative potency of extracts on rat intestinal α-glucosidase activity *in vitro* as well as hypoglycemic effects *in vivo*. Green, oolong, and black tea bags were extracted in hot water and the remaining tea pomace were dried and further extracted in 70% ethanol. The extracts were determined for intestinal rat α-glucosidases activity, radical scavenging activity, and total phenolic content. The postprandial glucose-lowering effects of TWE and TPE of green and black tea were assessed in male Sprague-Dawley (SD) rats and compared to acarbose, a known pharmacological α-glucosidase inhibitor. The IC_50_ values of all three tea extracts against mammalian α-glucosidase were lower or similar in TPE groups than those of TWE groups. TWE and TPE of green tea exhibited the highest inhibitory effects against α-glucosidase activity with the IC_50_ of 2.04 ± 0.31 and 1.95 ± 0.37 mg/mL respectively. Among the specific enzymes tested, the IC_50_ values for TWE (0.16 ± 0.01 mg/mL) and TPE (0.13 ± 0.01 mg/mL) of green tea against sucrase activity were the lowest compared to those on maltase and glucoamylase activities. In the animal study, the blood glucose level at 30 min after oral intake (0.5 g/kg body wt) of TPE and TWE of both green and black tea was significantly reduced compared to the control in sucrose-loaded SD rats. The TPE of all three teas had significantly higher phenolic content than those of the TWE groups, which correlated strongly with the DPPH radical scavenging activity. This is the first report of tea pomace extract significantly inhibits intestinal α-glucosidase, resulting in delayed glucose absorption and thereby suppressed postprandial hyperglycemia. Our data suggest that tea pomace-derived bioactives may have great potential for further development as nutraceutical products and the reuse of otherwise biowaste as valuable bioresources for the industry.

## 1. Introduction

Type 2 diabetes mellitus (T2DM) is a metabolic disorder of glucose and fat metabolism and is strongly associated with increased cardiovascular disease risk and oxidative damages [1]. Postprandial hyperglycemia has been recognized as an early defect in patients with T2DM and is due, primarily, to impaired insulin secretion or insulin resistance, as well as environmental factors such as diet and exercise [2]. Controlling postprandial plasma glucose levels is thus critical in the early treatment of diabetes and is one of the primary targets for anti-diabetic drugs.

As plasma blood glucose levels after a meal is related to the amount and digestion rate of consumed carbohydrate, one therapeutic approach for managing postprandial hyperglycemia is to retard digestion and subsequent absorption of dietary complex carbohydrates [3]. In humans, dietary starch is primarily digested in the small intestine by the action of α-amylase, generating disaccharides and oligosaccharides, which are further hydrolyzed by α-glucosidase to yield individual monosaccharides [4]. α-glucosidase inhibitors have been shown to be effective in suppressing postprandial hyperglycemia by limiting glucose absorption and the resulting insulin response [5]. Currently, acarbose is one of the widely used α-glucosidase inhibitors to treat patients with T2DM and also to reduce vascular complications such as retinopathy and neuropathy [6]. However, synthetic α-glucosidase inhibitors such as acarbose are often reported to be associated with gastrointestinal side effects including abdominal pain, flatulence, and diarrhea due to bacterial fermentation of the undigested carbohydrate in the colon [7].

Recent studies suggest that intestinal α-glucosidase and α-amylase inhibitors from natural sources such as plant-based foods could be as effective as synthetic drugs with lesser undesirable side effects [8]. In particular, polyphenolic compounds present in medicinal plants have many health-promoting activities and have been shown to exert intestinal α-glucosidase and α-amylase inhibitory effects *in vitro* [9,10,11], suggesting their potential use for controlling postprandial blood glucose management [12,13].

Tea is among the most commonly consumed and popular beverages worldwide [14]. Derived from the leaves of the plant, *Camellia sinesis*, there are three major types of tea depending on the degree of fermentation [14]. Green tea is an unfermented product containing relatively large amounts of polyphenolic compounds compared to that of oolong tea and black tea, where oolong tea is partially fermented and black tea is fully fermented [14]. Several *in vitro* and *in vivo* studies have shown that tea consumption as a beverage or use of tea extracts has anti-hyperglycemic and antioxidative effects *in vitro* study [15], in animal models and humans [16,17,18]. Furthermore, green tea supplementation has also been reported to ameliorate insulin resistance in fructose-fed rat model [19]. While there are reports on the beneficial health effects of tea consumption in diabetes, it is not known whether the tea pomace, the main biowaste from tea processing, has potential anti-diabetic properties.

Pomace is the main biowaste byproduct generated in the beverage-making industry that can accumulate rapidly to large amounts, leading to waste management issues. Pomace contains many useful bioactive compounds such as polyphenolic compounds, organic acids, and edible fibers, which can be recycled and reused [20]. Several studies showed that pomace obtained from natural sources has many beneficial health effects such as grape pomace on anti-hyperglycemic effects in diabetic mice [20], pear pomace on anti-adipogenic effects [21], and blueberry pomace on improved metabolic parameters associated with metabolic syndrome [22]. To the best of our knowledge, this is the first report on the effect of green and oolong tea pomace on the inhibition of rat intestinal α-glucosidase activity consistent with *in vivo* hypoglycemic effects in rats. Although there is one recent study that showed the inhibitory effect of black tea pomace on α-glucosidase activity, the *in vivo* glucose-lowering activity was not evaluated in this study [23].

Therefore, this study was aimed to evaluate and compare anti-diabetic potential of tea pomace extract (TPE) and tea water extract (TWE) by determining their *in vitro* inhibitory activities on α-glucosidase including sucrase, maltase, and glucoamylase. *In vivo* study was also performed to investigate the effect of TPE and TWE of green and black tea on postprandial glycemic response and compared their effects to a pharmacological α-glucosidase inhibitor, acarbose, in sucrose-fed Sprague-Dawley rats. In addition, we measured total phenolic content and DPPH radical scavenging activity in TPE and TWE of green, oolong, and black tea to determine a correlative relationship.

## 2. Results and Discussion

### 2.1. Rat α-Glucosidase Inhibitory Activity

**Figure 1:**
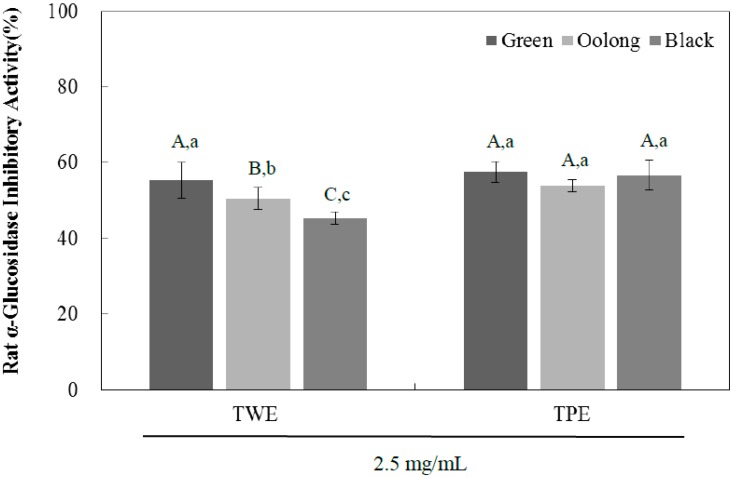
The inhibitory effects of tea water extract (TWE) and tea pomace extract (TPE) of green, oolong, and black tea at a concentration of 2.5 mg/mL on rat small intestinal α-glucosidase activity. The α-glucosidase activity was determined by measuring *p*-nitrophenol released from pNPG at 404 nm. Results are expressed as mean ± SD from three independent experiments in triplicate. Different corresponding letters indicate significant differences at *p* < 0.05 by Duncan’s test. ^A−C^ The first letters in uppercase are different among the types of tea within same extraction method and ^a−c^ the second letters in lower-case indicate significant differences between TWE and TPE groups.

In order to investigate the bioactivity of tea pomace after water extraction, enzyme inhibitory activities of tea water extract (TWE) and tea pomace extract (TPE) of green, oolong, and black tea against rat intestinal α-glucosidase were evaluated using 4-nitrophenyl α-d-glucopyranoside (*p*NPG) method as described in Materials and Methods [4]. Although yeast α-glucosidase has been used often to screen natural compounds for their anti-diabetic potential, results from yeast α-glucosidase may not provide us with any practical information. Therefore, we used a rat small intestinal α-glucosidase enzyme, which is more relevant to mammalian systems in the present study. As shown in Figure 1, the inhibitory potency of green tea in the TWE group at a concentration of 2.5 mg/mL was significantly higher (57%) compared to oolong tea (50%), both of which were significantly higher than black tea (45%). The TPE of all teas were as potent as TWE of green tea in inhibiting the enzyme activity and there were no statistical differences among TPE groups. Table 1 shows the *in vitro* half-maximal inhibitory concentration (IC_50_) of TPE and TWE of green, oolong, and black tea on rat intestinal α-glucosidase activity. The IC_50_ values for the TPE of green tea appear to be the lowest (1.95 ± 0.37 mg/mL) and the TPE groups in general exhibited higher inhibitory activities on rat small intestinal α-glucosidase than those of TWE groups. These results indicate that the inhibitory potency of tea pomace extracts is comparable to that of the TWE groups, which is likely due to the presence of high phenolic bioactive compounds after hot water extraction. Thus, tea pomace, the primary byproduct from tea processing in the tea industry, has great potential to be developed as inexpensive nutraceutical products for the management of hyperglycemia with reduced side effects.

**Table 1:** Half maximal inhibitory concentration (IC_50_) of tea extracts on rat intestinal α-glucosidase activity.

	IC_50_ (mg/mL)			
		Green Tea	Oolong Tea	Black Tea
**Rat intestinal α-glucosidase**	**TWE**	2.04 ± 0.31 ^C,cd^	2.33 ± 0.25 ^B,b^	2.73 ± 0.15 ^A,a^
	**TPE**	1.95 ± 0.37 ^B,d^	2.22 ± 0.52 ^A,bc^	2.14 ± 0.21 ^AB^^,b−d^

Different corresponding letters indicate significant differences at *p* < 0.05 by Duncan’s test. ^A–C^ The first letters in uppercase are different among the types of tea within same extraction method and ^a–d^ the second letters in lower-case indicate significant differences among all samples. TWE: tea water extract, TPE: tea pomace extract.

### 2.2. Sucrase, Maltase, and Glucoamylase Inhibition Assay

**Figure 2:**
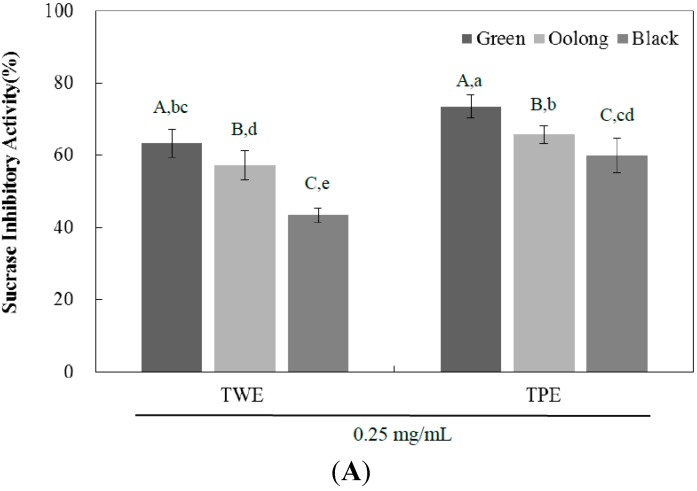

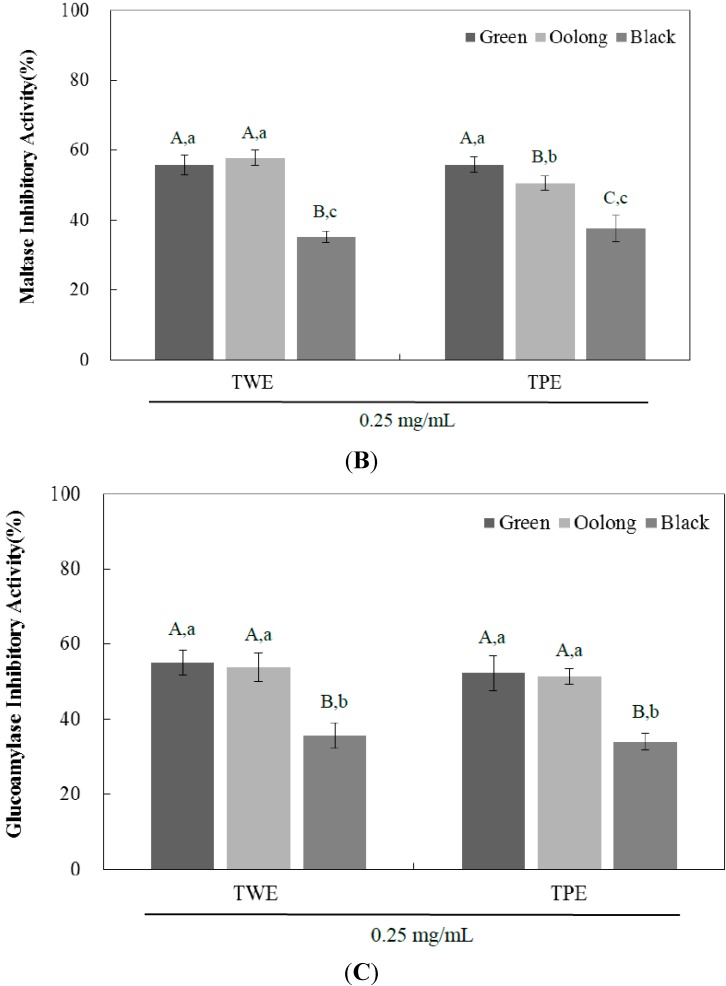
The inhibitory effects of tea water extract (TWE) and tea pomace extract (TPE) of green, oolong, and black tea at a concentration of 0.25 mg/mL on rat small intestinal sucrase (**A**); maltase (**B**); and glucoamylase (**C**) activities. The absorbance was measured at 530 nm and the results were expressed as mean ± SD with three independent experiments in triplicate. Different corresponding letters indicate significant differences at *p* < 0.05 by Duncan’s test. ^A−C^ The first letters in uppercase are different among types of tea within same extraction method and ^a−e^ the second letters in lowercase indicate significant differences among all samples.

The α-glucosidase inhibitors interfere with enzymatic actions in the brush-border of the small intestine, inhibiting the liberation of d-glucose from oligosaccharides and disaccharides. This results in delayed rate of glucose absorption, reducing postprandial plasma glucose levels [4]. To determine whether the inhibitory activity is specific, we examined the effect of TPE and TWE of all three teas on individual glucosidase enzymes such as sucrase, maltase, and glucoamylase. At the concentration of 0.25 mg/mL, the percentage intestinal sucrase inhibitory activity of TWE and TPE of green tea was significantly higher than those of oolong tea, followed by those of black tea as shown in Figure 2A and Table 2. The sucrase inhibitory potency of TWE and TPE of all teas were higher than that of maltase and glucoamylase (Figure 2B,C and Table 2). For maltase and glucoamylase, the percentage inhibitory activity of both TWE and TPE of green and oolong tea were similar but significantly different compared to those of black tea. Taken together, the inhibitory effect of tea pomace extracts on intestinal α-glucosidase activity was comparable to the tea water extracts, which may be attributable to their inhibitory activities on sucrase, maltase, and glucoamylase. These data suggest that tea pomace extract may act as a potent and effective inhibitor of α-glucosidase with a stronger inhibition on sucrase activity compared with the activities of maltase and glucoamylase.

**Table 2:** Half maximal inhibitory concentration (IC_50_) of tea water extract (TWE) and tea pomace extract (TPE) on rat small intestinal sucrase, maltase, and glucoamylase activity

		IC_50_ (mg/mL)		
		Green Tea	Oolong Tea	Black Tea
**Sucrase**	**TWE**	0.16 ± 0.01 ^C,c^	0.19 ± 0.01 ^B,b^	0.31 ± 0.04 ^A,a^
	**TPE**	0.13 ± 0.01 ^C,d^	0.16 ± 0.01 ^B,c^	0.19 ± 0.01 ^A,b^
**Maltase**	**TWE**	0.22 ± 0.01 ^B,d^	0.22 ± 0.01 ^B,d^	0.37 ± 0.03 ^A,a^
	**TPE**	0.22 ± 0.01 ^C,d^	0.26 ± 0.01 ^B,c^	0.35 ± 0.03 ^A,b^
**Glucoamylase**	**TWE**	0.22 ± 0.01 ^B,b^	0.23 ± 0.01 ^B,b^	0.37 ± 0.05 ^A,a^
	**TPE**	0.24 ± 0.02 ^B,b^	0.25 ± 0.01 ^B,b^	0.38 ± 0.01 ^A,a^

Different corresponding letters indicate significant differences at *p* < 0.05 by Duncan’s test. ^A−C^ The first letters in uppercase are different among the types of teas within same extraction method and ^a−d^ the second letters in lowercase indicate significant differences among all samples.

### 2.3. Total Phenolic Contents of Tea Extracts

To determine the differences in phenolic profiles, the total phenolic content of the tea extracts was measured and the results were expressed as gram gallic acid equivalent (GAE) per 100 gram dried weight of extracts (Table 3). The total phenolic contents of TPE groups of all tea extracts were higher than those of TWE groups. Among the TPE groups, green tea extract had the highest content of total phenolic compounds (63.94 ± 1.66), and black tea extract had the lowest content (58.24 ± 0.78), which was still comparably higher than the TWE of all three teas. Table 3 shows the total phenolic content of the samples with the TPE groups having significantly higher total phenolic content than TWE groups. Among the tea samples, green (TWE: 48.90 and TPE: 63.94 mg/mL) and oolong (TWE: 48.65 and TPE: 61.82 mg/mL) had similar amounts of phenolic contents but contained more phenolic contents compared to black (TWE: 40.14 and TPE: 58.24 mg/mL) tea extracts.

**Table 3:** Total phenolic content of tea water extract (TWE) and tea pomace extract (TPE) of green, oolong, and black tea.

		Total Phenolic Contents		
		Green Tea	Oolong Tea	Black Tea
**g/100 g dried wt of extract**	**TWE**	48.90 ± 1.14 ^A,d^	48.65 ± 1.24 ^A,d^	40.14 ± 1.04 ^B,e^
**(GAE)**	**TPE**	63.94 ± 1.66 ^A,a^	61.82 ± 0.38 ^A,b^	58.24 ± 0.78 ^B,c^

Different corresponding letters indicate significant differences at *p* < 0.05 by Duncan’s test. ^A,B^ The first letters in uppercase are different among the types of tea within same extraction method and ^a−e^ the second letters in lowercase indicate significant differences among all samples.

### 2.4. Antioxidant Activity by DPPH Radical Scavenging Assay

**Figure 3:**
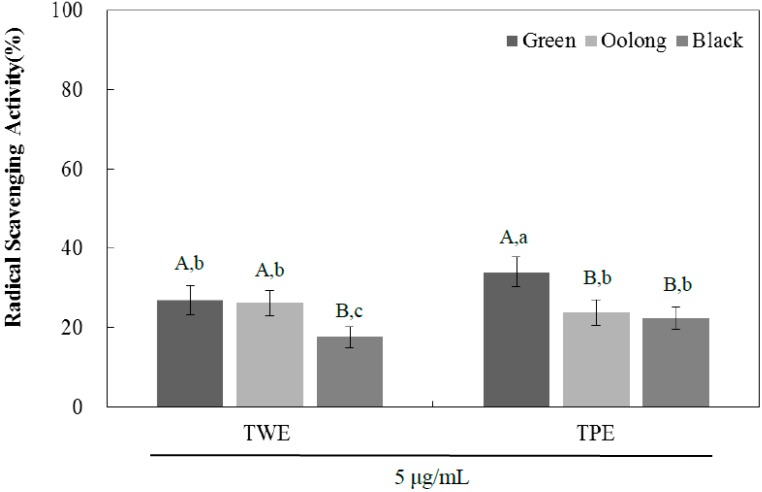
The DPPH radical scavenging activities of tea water extract (TWE) and tea pomace extract (TPE) of green, oolong, and black tea at a concentration of 5.0 µg/mL. The absorbance was measured at 517 nm, and the results were expressed as mean ± SD with three independent experiments in triplicate. Different corresponding letters indicate significant differences at *p* < 0.05 by Duncan’s test. ^A,B^ The first letters in uppercase are different among types of tea within same extraction method and ^a−c^ the second letters in lowercase indicate significant differences among all samples.

**Figure 4:**
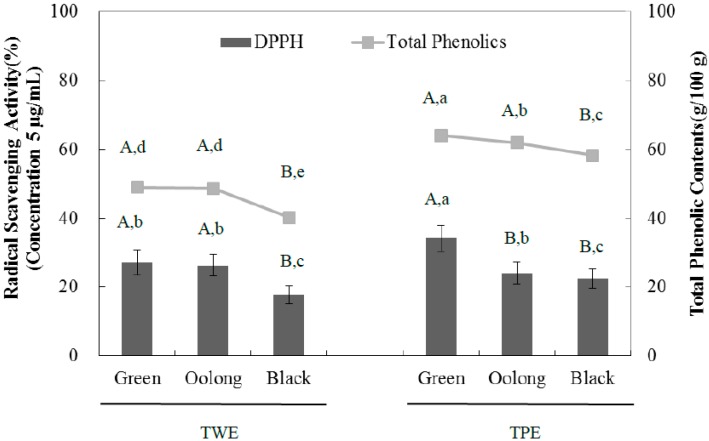
Comparison of DPPH radical scavenging activities and total phenolic content of tea water extract (TWE) and tea pomace extract (TPE) of green, oolong, and black tea at concentration 5.0 µg/mL concentration, The absorbance was measured at 517 nm for DPPH, and at 725 nm for total phenolic content. The results were expressed as mean ± S.D with three independent experiments in triplicate. Different corresponding letters indicate significant differences at *p* < 0.05 by Duncan’s test. ^A,B^ The first letters in uppercase are different among types of tea within the same extraction method and ^a−e^ the second letters in lowercase indicate significant differences among all samples.

The antioxidant activity was evaluated using the DPPH radical scavenging potential for TPE and TWE of green, oolong, and black tea (Figure 3). At the concentration of 5 µg/mL, all samples tested had antioxidant capacity with the TWE of green and oolong tea exhibiting significantly higher activity than black tea. However, the antioxidant activity of the TPE of green tea however was significantly higher than that of green tea and black tea. The IC_50_ values were consistent with the DPPH radical scavenging activities for all samples examined (Table 4). In order to evaluate a linear relationship between the observed antioxidant activity and total phenolic content, Pearson’s correlation coefficient was determined and we observe a strong correlation between antioxidant activity and total phenolic content. The correlation coefficient values of TWE and TPE are 0.994 and 0.857, respectively (Figure 4). A plant-based diet is recommended to prevent many chronic diseases including diabetes, and its health-promoting properties are generally attributed to antioxidant phenolic compounds. Tea is known to have high flavonoid content, primarily catechins such as (−)-epigallocatechin gallate (EGCG), (−)-epigallocatechin (ECG), (−)-gallocatechin (GC), and (+)-catechin (C). Although further study is needed to determine if specific flavonoids are responsible for the observed biological activities, the inhibitory effect on α-glucosidase activity is likely mediated by the contribution of several or multiple bioactive compounds of the tea extracts, that could act alone or in synergy. In addition, the pomace extracts of all three teas contained significantly higher amounts of total phenolic contents than those of the aqueous extracts, suggesting significant amounts of hydrophobic compounds present in the tea pomace extracts. These compounds could also be potentially important for the inhibitory effects of the tea pomace extracts on α-glucosidase activity as well as blood glucose-lowering effects.

**Table 4:** Half maximal inhibitory concentration (IC_50_) of tea water extract (TWE) and tea pomace extract (TPE) of green, oolong, and black tea on the DPPH radical scavenging activity.

		IC_50_ (µg/mL)		
		Green	Oolong	Black
**DPPH**	**TWE**	15.33 ± 2.61 ^B,b^	16.14 ± 1.25 ^B,b^	20.57 ± 0.78 ^A,a^
	**TPE**	9.21 ± 1.86 ^B,c^	16.06 ± 1.17 ^B,b^	18.05 ± 0.50 ^A,a^

Different corresponding letters indicate significant differences at *p* < 0.05 by Duncan’s test. ^A,B^ The first letters in uppercase are different among types of tea within the same extraction method and ^a−c^ the second letters in lowercase indicate significant differences among all samples.

### 2.5. Sucrose Loading Test

**Figure 5:**
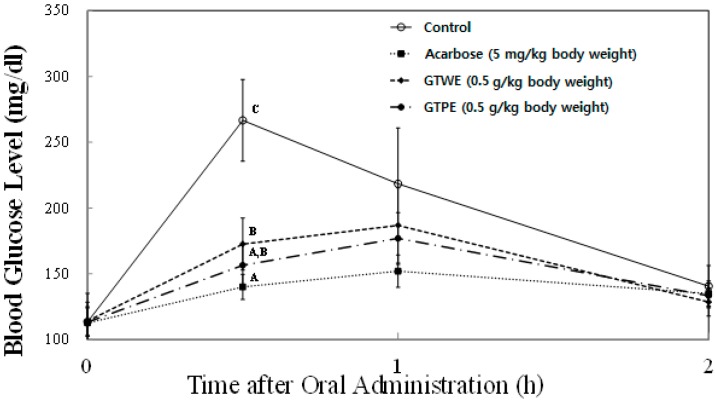
Comparison of postprandial blood glucose-lowering effects of green tea water extract (GTWE) and green tea pomace extract (GTPE) in a sucrose loading test. After a 24 h fast, six-week-old, male SD rats were orally administered with sucrose solution (2.0 g/kg) with or without samples (GTWE, GTPE, and a positive control: acarbose). Each point represents Mean ± SD (*n* = 5). ^A−C^ Different corresponding symbols indicate significant differences at *p* < 0.05 by Duncan’s test.

**Figure 6:**
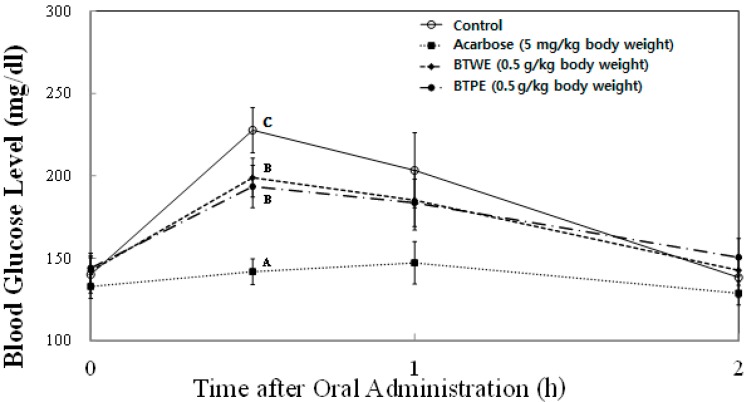
Comparison of postprandial blood glucose-lowering effects of black tea water extract (BTWE) and black tea pomace extract (BTPE) in sucrose loading test. After a 24 h fast, six-week-old, male SD rats were orally administered with sucrose solution (2.0 g/kg) with or without samples (BTWE, BTPE. and a positive control: acarbose). Each point represents Mean ± SD (*n* = 5). ^A-C^ Different corresponding symbols indicate significant differences at *p* < 0.05 by Duncan’s test.

In order to further confirm the *in vitro* inhibition of sucrase activity, the time courses of plasma glycemic response were measured at 0, 30, 60, and 120 min after sucrose-loading (2.0 g/kg body wt) in SD rats. Since we observed the highest activity with green tea extract, and black tea is the most consumed type of tea, we evaluated the blood glucose-lowering effects of these two tea types in animal experiments. *In vivo* plasma glucose-lowering effects of TPE and TWE of green and black tea at the concentration of 0.5 mg/kg body wt were compared with the control group (no treatment) and a known pharmacological α-glucosidase inhibitor, acarbose (5 mg/kg body wt) as a positive control (Figure 5 and Figure 6). In SD rats treated with both TPE and TWE of green tea significantly decreased plasma glucose level (156.5 ± 12.0 mg/dL and 172.6 ± 14.0 mg/mL, respectively) compared with the control group (266.6 ± 31.1 mg/dL) at 30 min following sucrose loading (Figure 5). However, there was no significant difference in the blood glucose level between TWE and TPE of green tea. As expected, acarbose significantly decreased blood glucose levels (140.0 ± 14.1 mg/dL) compared to the control and TWE of green tea. Meanwhile, SD rats treated with both black tea TPE (193.5 ± 12.9 mg/dL) and TWE (198.9 ± 11.7 mg/dL) showed significant decreases in plasma glucose levels, although there were no significant differences between TPE and TWE (Figure 6). These results strongly indicate the potency of tea and tea pomace extracts to significantly reduce plasma glucose excursion in response to diets high in sucrose. 

## 3. Materials and Methods

### 3.1. Materials

Green, black, and oolong tea (Granum Inc., Seattle, WA, USA) were purchased from the local supermarket (Amherst, MA, USA). Corn starch, casein, vitamin mix, mineral mix, calcium phosphate and sodium chloride were purchased from Raon Bio (Yongin, Korea). Blood glucose analyzer was purchased from Caresens (I-SENS, Anyang, Korea). 2,2-Diphenyl-1-picrylhydrezyl was purchased from Alfa Aesar. (Ward Hill, MA, USA). Folin-Ciocalteu phenol reagent, rat intestinal acetone powders, 4-nitrophenyl α-d-glucopyranoside, *o*-Dianisidine dihydrochloride, glucose oxidase/peroxidase reagents were purchased from Sigma-Aldrich Co. (St. Louis, MO, USA). Unless noted otherwise, all chemicals were purchased from Fisher Scientific Company Co. (Bridgewater, NJ, USA).

### 3.2. Preparation of Tea and Tea Pomace Extracts

Ten g of each selected green, black, and oolong tea bags were extracted in 1000 mL of water at 95 °C for 10 min. After hot water extraction, the resulting tea bags were dried at 50 °C for 24 h (tea pomace). Ten g of tea pomace were then further extracted in 200 mL of 70% ethanol at 25 °C for 2 h. The tea water extract and tea pomace extract were then filtered through Whatman No. 4 filter paper using a vacuum flask. After evaporating the organic solvent, the resulting tea extracts were dried at 50 °C overnight, reconstituted in dimethyl sulfoxide (DMSO), and stored at −20 °C until analysis.

### 3.3. Rat α-Glucosidase Inhibition Assay

Rat intestinal α-glucosidase activity was determined using the substrate *p*-nitrophenyl-α-d-glucopyranoside (pNPG) as previously described by Kwon *et al.* [24] with a slight modification. A total of 3 g of rat-intestinal acetone powder was suspended in 9 mL of 0.9% saline, and the suspension was vortexed for 30 s at 4 °C. It was then centrifuged (13,000× *g*, 15 min, 4 °C), and the resulting supernatant was used for the assay. Sample solution (50 μL) and 0.1 M phosphate buffer (pH 6.9, 100 μL) containing rat intestinal α-glucosidase solution (1.0 U/mL) were incubated at 37 °C for 10 min. After the incubation, 5 mM pNPG solution (50 μL) in 0.1 M phosphate buffer (pH 6.9) were added to each well at timed intervals. The reaction mixtures were further incubated at 37 °C for 30 min. Absorbance was measured at 405 nm and compared to a control which had 50 μL of buffer solution in place of the extract by Micro-Plate Reader (Molecular devices; Sunnyvale, CA, USA). The rat α-glucosidase inhibitory activity was expressed as percent inhibition and was calculated as follows:

$$\text{Inhibition}\;(\text{\%})=[\frac{{∆\;A\;}_{\text{Control}\;405}-{∆\;A\;}_{\text{Extract}\;405}}{{∆\;A\;}_{\text{Control}\;405}}]\times 100$$

### 3.4. Sucrase, Maltase, Glucoamylase Inhibition

The enzyme solution prepared from rat intestinal acetone powder was used to measure the activity of the small intestinal maltase, sucrase, and glucoamylase enzymes. Rat intestinal acetone powder (3.0 g) was suspended in 9 mL of 0.9% saline solution, and the suspension was sonicated twelve times for 30 s at 4 °C. After centrifugation (13,000× *g*, 15 min, 4 °C), the resulting supernatant was used for the assay. Maltase, sucrase, and glucoamylase inhibitory activity were assayed by modifying a method developed by Dahlqvist [25]. The inhibitory activity was determined by incubating a solution of an enzyme (50 μL), 0.1 M phosphate buffer (pH 7.0, 100 μL) containing 0.4 mg/mL sucrose or maltose or 1% soluble starch, and a solution (50 μL) with various concentrations of sample solution (between 0.025 and 0.25 mg/mL) at 37 °C for 30 min. The reaction mixture was heated in a boiling water bath to stop the reaction for 10 min, and then the amount of liberated glucose was measured by the glucose oxidase method [26]. The inhibitory activity was calculated from the formula as follows:

$$\text{Inhibition}\;(\text{\%})=[\frac{{∆\;A\;}_{\text{Control}\;530}-{∆\;A\;}_{\text{Extract}\;530}}{{∆\;A\;}_{\text{Control}\;530}}]\times 100$$

### 3.5. Total Phenolic Contents

Total phenolic contents were determined following the procedure previously described by Ranilla *et al.* [27]. One mL extract was mixed with 95% ethanol and 5 mL distilled water in test tubes. To each sample, 50% (*v*/*v*) Folin-Ciocalteu phenol reagent was added into the mixture and allowed reaction at room temperature for 5 min. One mL 5% Na_2_CO_3_ was then added to the reaction mixture and stored in dark place for 60 min. The absorbance was measured at 725 nm and compared to a control, which had 0.5 mL of water in place of the extract using Micro-Plate Reader. The values were converted to total phenolics and were expressed in gallic acid equivalent (g/100 g dried weight of extract). Standard curve was established using various concentrations of gallic acid in ethanol.

### 3.6. Antioxidant Activity by 2,2-Diphenyl-1-picrylhydrezyl Radical (DPPH) Inhibition Assay 

The antioxidant capacity was determined by DPPH radical scavenging method as described by Kwon *et al.* [9] with some modifications. A sample solution (10 µL) was mixed with 190 μL of DPPH solution in 95% ethanol and the mixture incubated for 30 min at 37 °C. Absorbance was measured at 517 nm by a Micro-Plate Reader and compared with controls which contained 10 µL of water in place of the extract. The DPPH radical scavenging activity was expressed as percent inhibition was calculated using the formula as follows:

$$\text{Inhibition}\;(\text{\%})=[\frac{{∆\;A\;}_{\text{Control}\;517}-{∆\;A\;}_{\text{Extract}\;517}}{{∆\;A\;}_{\text{Control}\;517}}]\times 100$$

### 3.7. Sucrose-Loading Test in Rats

All animal procedures were approved by the Institutional Animal Care and Use Committee (IACUC) of Hannam University (Approval number: HNU2014-0019). Five week-old male Sprague-Dawley (SD) rats were purchased from Joongang Experimental Animal Co. (Seoul, Korea) and fed with standard diet (Samyang Diet Co., Seoul, Korea) and with water *ad libitum* for one week. The rats were housed in a ventilated room at 25 ± 2 °C with 50% ± 7% relative humidity, and under an alternating 12-h light/dark cycle. After 1 week of acclimation, 20 SD male rats (180~200 g) were assigned to 4 groups with similar body weight distribution in each group and fasted for 24 h. Group 1 received sucrose (2.0 g/kg body wt) as the control, group 2 was co-administered with sucrose (2.0 g/kg body wt) and acarbose (5.0 mg/kg body wt), group 3 was co-administered with sucrose (2.0 g/kg body wt) and green tea water extract (GTWE, 0.5 g/kg body wt), and group 4 was co-administered with sucrose (2.0 g/kg body wt) and green tea pomace extract (GTPE, 0.5 g/kg body wt). For black tea experiments (Figure 6), groups 1 and 2 were the same, group 3 was co-administered with sucrose (2.0 g/kg body wt) and black tea water extract (BTWE, 0.5 g/kg body wt) and group 4 was co-administered with sucrose (2.0 g/kg body wt) and black tea pomace extract (BTPE, 0.5 g/kg body wt). The blood samples were taken from the tail and its blood glucose level was measured periodically at 0, 0.5, 1, and 2 h time intervals. The glucose level in blood was determined by glucose oxidase method and compared with that of the control group, which had not taken the inhibitors.

### 3.8. Statistical Analysis

All data are presented as mean ± SD for each assay. Statistical analysis was carried out using the statistical package SPSS 10 (Statistical Package for Social Science, SPSS Inc., Chicago, IL, USA) program and significance of each group was verified with One-way analysis of variance (ANOVA) followed by the Duncan’s multiple range test and the Student’s *t*-test for comparison of means. *p*-Values of less than 0.05 were considered to be statistically significant.

## 4. Conclusions

Postprandial hyperglycemia has been identified as a major risk factor for cardiovascular complications associated with T2DM, and thereby recognized as a key therapeutic target in the treatment of T2DM. Our data presented in this study strongly suggest that extracts of tea pomace, a waste byproduct of tea processing, can be a significant source of bioactive phenolic compounds that exert antioxidant and postprandial blood glucose-lowering effects. Based on our obtained results, a potential mechanism may involve the inhibition of α-glucosidase enzyme in the brush border of small intestine, similar to that of acarbose, a drug currently prescribed to treat postprandial hyperglycemia in patients with T2DM. Although further work is still needed to identify specific anti-hyperglycemic components in the TPE and evaluate pharmacological and biological effects, our *in vitro* and *in vivo* data offer a biochemical rationale for further animal and clinical studies. These results also provide the basis for developing valuable alternatives to synthetic drugs to manage glycemic control from tea pomace, the widely wasted main byproduct in the tea making industry.
